# A covert cortical ensemble for learned fear suppression

**DOI:** 10.1038/s41386-024-01991-3

**Published:** 2024-09-16

**Authors:** Rebecca M. Shansky, Eliza M. Greiner

**Affiliations:** Dept. of Psychology, Northeastern University, Boston, MA USA

**Keywords:** Fear conditioning, Extinction

People with Post-Traumatic Stress Disorder (PTSD) suffer from a spectrum of symptoms that range from disruptions in sleep, cognition, and mood to full-blown panic attacks elicited by reminders of their trauma. Reducing the frequency and magnitude of these episodes is a primary goal for clinicians who treat PTSD patients, and doing so requires teaching the brain to actively suppress an emotional response that environmental cues might trigger. This is most commonly done through a process called “exposure therapy,” in which a patient is intentionally presented with the sensory stimuli that are most likely to remind them of the traumatic event that precipitated their diagnoses. By repeatedly experiencing these cues in a safe, controlled environment, the patient learns that the cues do not always signal danger, and the emotional responses that they once elicited will abate. Despite its widespread use in the clinic, however, the effects of exposure therapy often lack staying power, posing a challenge for both clinical and preclinical researchers alike [1]. In this issue of *Neuropsychopharmacology*, work from the latter realm by Bayer et al. [2] offers novel insight into a behind-the-scenes neural process that could promote more long-term success in suppressing cue-induced fear responses.

The learning principles on which exposure therapy is based have roots in Pavlovian fear conditioning and extinction [3], classic behavioral paradigms in which an animal (usually a rodent) first learns to associate an auditory tone with an aversive foot shock, thus producing a conditioned fear response to the tone, traditionally measured as freezing behavior. This response can then be suppressed or “extinguished” through repeated presentation of the tone alone, as the animal learns that the tone no longer predicts the foot shock. Several decades of work have shown that a sub-region of the medial prefrontal cortex known as the infralimbic cortex (IL) is critical to extinction processes, but it was generally thought that the IL did not come online until extinction itself began.

Prior work by Bayer and Bertoglio [4], however, found that the IL might in fact be engaged earlier than was previously appreciated. Specifically, pharmacological inactivation of the IL immediately after fear conditioning resulted in elevated freezing and impaired extinction when animals were drug-free, suggesting that the consolidation of a fear memory may stimulate a neural ensemble in the IL that, while seemingly unnecessary for learning the association itself, is later recruited to facilitate extinction.

In the current manuscript, the authors pushed this idea further by giving the IL a pharmacological boost with the GABA receptor antagonist picrotoxin, which induces neural excitation. Picrotoxin administered immediately after fear conditioning produced a dramatic acceleration of extinction 48 h later, an enhanced suppression of fear that importantly persisted in an extinction retrieval test the following day. Additional experiments demonstrated that these effects were not restricted to the post-conditioning consolidation window, as the same reduction in freezing was observed in animals that received intra-IL picrotoxin 24 h or even 13 days after fear conditioning. Finally, the authors demonstrated that despite this flexibility in when IL stimulation could be effective in facilitating extinction, the post-conditioning consolidation period is still a critical time point, because blocking protein synthesis in the IL with anisomycin during this window prevented picrotoxin’s effects.

Together, the results here paint a provocative picture: that during conditioned fear consolidation, while other structures within the brain (e.g. thalamus, amygdala, hippocampus, and prelimbic cortex) are working to form the associative memory that will *enable* a conditioned response when the animal encounters the tone in the future [5], the infralimbic cortex is covertly orchestrating a counter-offensive, to be deployed when that tone is no longer followed by a shock. The authors speculate that this process reflects the formation of a secondary “inhibitory memory,” and that pharmacological stimulation of this ensemble may essentially prime the IL to suppress conditioned freezing, thus facilitating extinction. Establishing the existence of such an ensemble on a cellular level using immediate early gene-based techniques (e.g. FosTRAP) or in vivo calcium imaging via mini-scopes will be an important next step in further defining the key time points and neural networks that ultimately determine how successful extinction-driven fear suppression will be.

From a translational perspective, what is especially exciting about this work is that the timing of IL stimulation with respect to extinction learning didn’t seem to matter – an isolated increase in IL activity both outside the fear memory consolidation window and yet weeks before extinction was sufficient to facilitate extinction learning and maintain fear suppression during a retrieval test. For PTSD patients undergoing exposure therapy, then, this could mean that the addition of interventions like deep brain stimulation or transcranial magnetic stimulation could give their brains a similar boost, thus promoting the efficacy and stability of exposure therapy. The major caveat here, based on data from the anisomycin experiment, is that the traumatic event needs to have created the “inhibitory memory” in the first place; without it, there is no ensemble to strengthen, and such interventions would have no effect. Building off this idea, it is tempting to wonder if PTSD patients—especially those for whom exposure therapy is less effective—represent a subset of trauma-exposed individuals in which this process has failed to occur.

Overall, the work here convincingly demonstrates that during fear conditioning, the IL is not simply an idle bystander, springing to action only once extinction commences. Instead, we see evidence for a system in which the IL may in fact be actively engaged by the fear conditioning experience, coordinating an ensemble of its own to ultimately help an individual adapt its behavior when the rules of the just-formed association change. Much work remains before the timing and mechanisms that drive the formation of this inhibitory ensemble (and its presumed re-activation by extinction) will come fully into focus, but this is nonetheless an exciting new finding with clear clinical implications.
